# Supplementary material for: Evidence of overfishing of geoduck clam Panopea globosa from a length-based stock assessment approach
Source: PeerJ. 2020 Jun 4;8:e9069. doi: 10.7717/peerj.9069 (PMC7276149; doi:10.7717/peerj.9069)
Supplement: Appendix A1 — Fishing area expressed as km2. [file peerj-08-9069-s001.docx]

**Appendix A1.** Changes and expansion of the fishing area (km^2^) of geoduck clam *Panopea globosa* in Puerto Peñasco, Sonora.

| **Time** | **Fishing area (km^2^)** |
| --- | --- |
| 2009 | 153,958 |
| 2010 | 180,483 |
| 2011 | 223,499 |
| 2012 | 210,729 |
| 2013 | 263,156 |
| 2014-2016 | 382,013 |
